# All-Rye-Based Transparent Composites for Bio-Based Food Packaging: Valorization of Rye Bran Cellulose Nanocrystals as Reinforcing Agents in Commercial Rye Arabinoxylan Films

**DOI:** 10.1021/acssuschemeng.5c04448

**Published:** 2025-08-27

**Authors:** Jon Trifol, Ana Isabel Mendoza, Rosana Moriana

**Affiliations:** † Gaiker Technology Center, Basque Research and Technology Alliance (BRTA), Parque Tecnológico de Bizkaia, Edificio 202, Zamudio 48170, Spain; ‡ Department of Fibre and Polymer Technology, School of Engineering Sciences in Chemistry, Biotechnology and Health, 7655 KTH-Royal Institute of Technology, Teknikringen 56, Stockholm 100 44, Sweden; § Research Group in Materials Technology and Sustainability (MATS), Department of Chemical Engineering, School of Engineering, University of Valencia, Avda. Universitat s/n, Burjassot 46100, Spain

**Keywords:** cereal bran, biomass valorization, nanocelluloses, hemicellulose-based nanocomposites, thermomechanical properties, gas barrier properties, optical properties, sustainable food packaging

## Abstract

This study presents, for the first time, the development of all-rye-based nanocomposite films for biobased food packaging by incorporating cellulose nanocrystals isolated from rye bran (RB-CNC) into a rye arabinoxylan (R-AX) matrix. The isolated RB-CNC exhibited high purity (>90% cellulose) and surface charge (−35.8 mV), together with an exceptional aspect ratio (∼61) and thermal stability (230 °C). Nanocomposite films were developed by incorporating RB-CNC at different loadings (5, 10, and 20 wt %) into commercial R-AX matrix. The resulting composites demonstrated significantly enhanced thermomechanical performance, competitive water vapor permeability, and excellent optical transparency, which are key properties required for packaging applications. Specifically, films with 10 wt % RB-CNC showed an 81% increase in Young’s modulus, a 49% enhancement in elongation at break, and a 98% rise in tensile strength compared to neat R-AX films. Optical transparency improved with RB-CNC content, with films achieving 95% transmittance and only 10% haze. All nanocomposites exhibited a thermal stability improvement exceeding 10 °C while retaining R-AX’s intrinsic water vapor barrier properties. These findings highlight the potential of RB-CNC as a high-performance, biobased reinforcement for hemicellulose-based biopolymers, paving the way for fully biobased, circular food packaging solutions.

## Graphical abstract

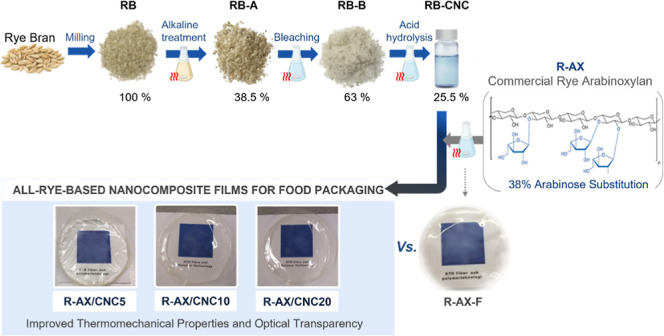

## 1 Introduction

The valorization of agro-industrial wastes and side-streams into biobased sustainable materials contributes to circular bioeconomy development fossil-based polymer wastes.^1^

Cereal grains are fundamental for global agriculture and human nutrition, with maize, wheat, rice, barley, oats, and rye ranking among the most widely consumed crops.^2^ According to the Food and Agriculture Organisation (FAO),^3^ global cereal production for the 2024/2025 season is projected to reach approximately 2.84 billion tonnes. An estimated 80% of the total cultivated grain volume is used for refined flour production, generating large amounts of cereal bran as a milling byproduct.^4^ Cereal bran consists of the outer grain layers (pericarp, cuticle, testa, and aleurone) and often retains residual starchy endosperm due to the inherent difficulties in achieving complete separation during milling.^5^ Its exact composition varies based on plant characteristics such as variety, kernel size, shape, maturity and outer layer thickness, as well as grain storage conditions, and milling methods.^2^ However, cereal bran is generally recognized as a rich source of dietary fiber, minerals, vitamins, and biocompounds. Despite containing these valuable compounds, cereal bran remains largely underutilized and is often relegated to low-value applications.^2^

Among cereal crops, rye (*Secale cereale* L.) is extensively cultivated across the Northern Hemisphere and ranks as the second most important grain for bread production after wheat.^5^ This cereal is typically processed into white flour by roller mills, generating between 17 and 36 million tonnes of rye bran annually during the past decade.^5,6^ Although its reported chemical composition in the literature varies,^7,8^ rye bran is predominantly composed of dietary fiber, (26.0–33.4% cellulose, 5.3–16.0% hemicellulose, and 3.3–13.0% lignin), along with starch (13.0–18.6%), protein (10.0–17.0%), and lipids (2.5–7.0%). These compositional attributes highlight its potential as a valuable feedstock for sustainable applications within the framework of biobased material development and circular economy principles. In this context, rye bran has been explored as a functional food additive,^9–11^ a substrate for fermentable sugars in ethanol production,^12–14^ and a natural feedstock for fuel pellet production.^15,16^ Additionally, rye bran has also been explored as a feedstock of bioactive compounds, such as alkylresorcinols^17^ and heteropolysaccharides,^18^ for plant and fruit protection. However, despite its cellulose content, the potential of rye bran for producing cellulose nanocrystals (CNC) remains unexplored, unlike other cereal brans such as barley,^19,20^ rice,^21–23^ and oat,^21,24^ from which CNC have been successfully extracted and characterized.

During the last decades, CNC have attracted increasing attention as reinforcing agents in high-performance polysaccharide-based nanocomposites for food packaging.^25^ CNC’s remarkable properties (including excellent transparency, superior mechanical strength, large specific surface area, effective resistance to grease, and low oxygen transmission rate under low relative humidity)^26^ make them highly promising for biobased and biodegradable packaging coatings and films. However, despite their advantages, CNC-based packaging materials face critical limitations, such as low flexibility, reduced thermal stability, and high moisture sensitivity, which contribute to increased water vapor permeability. To unlock the full potential of CNC-based nanocomposites for sustainable food packaging, it is essential to address these limitations by optimizing their integration with compatible polysaccharide matrices, ensuring that the resulting materials possess the necessary properties for effective packaging materials, such as robust mechanical strength, flexibility, transparency, and resistance to both oxygen and water vapor.

Arabinoxylan (AX)-based materials have been extensively studied for edible and biodegradable packaging film applications due to their natural origin, intrinsic functionality, and biodegradability.^27–31^ AX, a hemicellulosic heteropolysaccharide abundant in cereal walls, is able to form dense macromolecular network films with low mobility, providing effective barrier properties against apolar substances such as oxygen and aromas.^27^ However, AX films often exhibit poor thermomechanical performance and elevated water vapor permeability (WVP), which may be attributed to their branched, amorphous structure and hydrophilic hydroxyl groups.^28^ To improve these properties, physical and/or chemical modifications are necessary.^28,30,31^ Among these, acetylation and plasticizer incorporation are commonly used to enhance flexibility, but these modifications usually affect gas barrier performance and reduce film toughness.^28,32^ To further improve the overall mechanical properties of AX films, nanocelluloses (such as CNC, cellulose nanofibrils (CNF), and bacterial nanocelluloses) have been proposed as reinforcing agents.^33–37^ CNC and bacterial nanocellulose reinforcements have been shown to enhance both strength and toughness without compromising flexibility^36,38^ of AX-based films, whereas CNF incorporation^33,39^ increases elongation but reduces toughness, while simultaneously improving thermal stability^34^ and reducing optical transparency.^34,39^ Nevertheless, the influence of CNC on the WVP and optical characteristics of AX-based composite^33,39^ films remains insufficiently studied, with no previous studies identified.

In this study, for the first time, rye bran is proposed as a raw material for CNC production, and the potential of these RB-CNC as reinforcement in rye arabinoxylan-based nanocomposite films for packaging is evaluated. The objectives of this work are to: (i) evaluate the structural and physicochemical transformations occurring during the conversion of rye bran from macro- to nanoscale dimensions; (ii) demonstrate the potential of CNC derived from rye bran as reinforcement in a commercial hemicellulose biopolymer, also extracted from rye, to develop fully rye-based polysaccharide nanocomposites; and (iii) evaluate the impact of CNC content on the thermomechanical, water vapor barrier, and optical characteristics of the designed polysaccharide-based films.

## 2 Materials and Methods

### 2.1 Materials and Chemicals

Rye (*S. cereale* L.) bran, obtained as a milling byproduct during cereal processing in Filipstad, Sweden, was kindly provided by Barilla Sverige AB. The bran was incubated at 40 °C for seven days and subsequently milled to a 20-mesh particle size using a Wiley mill Acm 82302 (Acmas Technocracy Pvt. Ltd., Germany). Rye arabinoxylan (>90%, CAS no.: 9049-27-1) from Megazyme International Ireland, with a molar mass of 386,000 g/mol, was used without further purification. All other chemicals were employed as received: H_2_SO_4_ (72% v/v, LabService AB, Sweden); NaOH (98%, reagent grade, Sigma-Aldrich); NaClO_2_ (80%, Sigma-Aldrich); CH_3_COONa (>99% ACS reagent Sigma-Aldrich) and CH_3_COOH (ACS reagent >99.8%, Sigma-Aldrich). The sugar references used, provided by Sigma-Aldrich, included d-glucose, d-xylose, and d-mannose (each with ≥99% purity; CAS nos. 50-99-7, 58-86-6, and 3458-28-4, respectively), along with l-rhamnose monohydrate (99%, CAS 6155-35-7), d-galacturonic acid monohydrate (97%, CAS 91510-62-2), and d-glucuronic acid (98%, CAS 6556-12-3).

### 2.2 Production of Cellulose Nanocrystals from Rye Bran

Cellulose nanocrystals (CNC) were isolated from rye bran following similar procedures previously described for other residual biomass sources, such as rice husks,^40^ pine cones,^41^ and forest residues.^42^ First, milled rye bran samples (RB) were subjected to alkaline and bleaching treatments to eliminate extractives, lignin, and hemicelluloses, facilitating cellulose fiber isolation. Subsequently, sulfuric acid hydrolysis was carried out to remove the amorphous domains of the cellulose fibres, obtaining rye bran CNC (RB-CNC).

#### 2.2.1 Alkaline and Bleaching Treatment

Following the methodology reported by Moriana et al.,^42^ RB (3%, w/v) “milled samples were treated three times with a 4.5 wt % NaOH solution at 80 °C for 2 h under mechanical stirring. Then, the alkaline samples were subjected to five bleaching treatments under 80 °C for 4 h under mechanical stirring. The solution used in the bleaching treatment consisted of equal parts of acetate buffer (2 M, pH 4.8), aqueous chlorite (1.7% w/v in water), and water. After each treatment (alkaline and bleaching), the biomass was filtered and washed with water until the chemicals were removed, and dried at room temperature”.

#### 2.2.2 Cellulose Nanocrystal Isolation

After bleaching, RB-CNC were isolated from RB-B samples using acid hydrolysis under similar conditions of solid and acid concentration but with a shorter reaction time than that described for rice husks.^40^ A screening evaluation indicated 20 min, rather than 45 min, as the optimal hydrolytic time. RB-B “(4 wt %) were treated with 65 wt % sulfuric acid (preheated) at 45 °C for 40 min under continuous stirring. The hydrolyzed material was washed with water and centrifuged at 25,000*g* for 20 min (Rotofix 32A Hettich Zentrifugen, Germany). The residue was water suspended and dialyzed against distilled water for several days, using a 6–8 kDa membrane (Spectra/Por 1, SpectrumLabs, Breda, The Netherlands). The resulting suspensions were sonicated for 10 min while cooling in an ice bath, centrifuged at 4500 rpm for 10 min to remove the higher particles and kept at 4 °C for further analyses”.

### 2.3 Preparation of Nanocomposite Films

Polysaccharide-based nanocomposite films of around 95 mg were prepared by film casting, using commercial rye arabinoxylan (R-AX) as the polymeric matrix and RB-CNC in varying amounts (5, 10, and 20 wt %) as reinforcing agents. An aqueous arabinoxylan solution was prepared (9 mg/mL) and stirred at 70 °C for approximately 3 h to form a homogeneous suspension. These suspensions were mixed with the different amounts of RB-CNC and stirred at 50 °C for 1 h. The resulting mixtures were then homogenized by an Ultra-Turrax at 9500 rpm for 15 min, cast into polystyrene Petri dishes (Ø = 55 mm), and oven-dried at 35 °C for 48 h. The nanocomposite films were then coded as R-AX/CNC5, R-AX/CNC10 and R-AX/CNC20, based on the percentage of RB-CNC loading in the composition. Additionally, pure arabinoxylan film was prepared as a control and labeled as R-AX-F.

### 2.4 Characterization

#### 2.4.1 Chemical Composition Analysis

Following previously published methodology,^40^ “the chemical composition (carbohydrate, Klason lignin, ash and extractive content)” of RB and its alkaline, bleached and hydrolyzed samples in their dried state was evaluated. “The content of the different samples was measured by using a Mettler Toledo HB43 moisture analyzer (Columbus, OH). Klason lignin was determined following the TAPPI standard T222 om-06 (TAPPI, 2006)”.^43^ The total amount of soluble extractives in a toluene/ethanol (2:1) mixture, followed by water and ethanol extraction of the RB, was evaluated following NREL’s LAP.^44^ Carbohydrate composition was analyzed via a traditional two-stage Saeman hydrolysis^45^ process, after which the “hydrolyzed monosaccharides were separated and quantified by HPAEC-PAD on an ICS-3000 system (Dionex Corp., Sunnyvale, CA)”. “The ash content of the samples was determined by thermogravimetric analysis (TGA)” using a Mettler Toledo TGA/DSC 1 STAR system operating under oxidative conditions, following a previously published method.^46^

#### 2.4.2 Gravimetric Yields

Alkaline, bleaching, and hydrolysis processing yields were defined as the ratio of the recovered fraction to the initial dry mass of the raw material for each treatment. For statistical reliability, a minimum of five replicates per sample was analyzed to calculate the mean values and standard deviations.

#### 2.4.3 Scanning Electron Microscopy

Surface morphology of the RB, RB-A, and RB-B was examined as indicated in previous studies,^42^ “using a JEOL JSM-5400 scanning electron microscope (SEM) (JEO Ltd., Japan) at 1 kV and 5 kV". Prior to imaging, samples were dried and sputtered with gold/palladium on a Cressington 208HR sputter coater, rendering a layer of 2.5 nm.

#### 2.4.4 Atomic Force Microscopy

The morphology and size distribution of RB-CNC, along with the surface topography of the composite films, were examined using atomic force microscopy (AFM) (Multimode V, Bruker, Santa Barbara, CA) following established methodologies previously reported for CNC characterization^40,47,48^ and polysaccharide film topography evaluation.^49^ Samples were prepared via layer-by-layer deposition on silicon wafers to obtain images of individualized RB-CNC. The wafer substrates were cleaned and plasma-treated (Harrick Scientific Corporation Model PDC 002) for 4 min at 30 W, then sequentially immersed in polyethyleneimine (0.45 mg/mL) for 3 min, followed by RB-CNC solution (0.00045 mg/mL) for 3 min before washing and drying. The deposited RB-CNC and film formulations were imaged in a dry state and in tapping mode, with RTESP silica cantilevers (Bruker) equipped with moderate stiffness (20–80 N/m) with 8 nm tip radius and working frequencies of 306–366 kHz. The particle diameters of the visualized RB-CNC were assessed by analyzing their height, which was assumed to correspond to their actual diameter, minimizing the overestimation of width measurements due to the tip geometry. Measurements of both average length and diameter were obtained by analyzing over 100 individual nanoparticles per sample.

#### 2.4.5 Dynamic Light Scattering

RB-CNC suspensions (0.06 mg/mL) were prepared and sonicated for 2 min at 20% pulse. The ζ-potential was then measured in triplicate at 20 °C using a Malvern Zetasizer ZEN3600 instrument (Malvern, UK).^47^

#### 2.4.6 Fourier Transform Infrared Spectroscopy

The samples (RB, RB-A, RB-B, and RB-CNC-F) were assessed by Fourier transform infrared (FTIR) spectroscopy (PerkinElmer Spectrum 100 FTIR) equipped with an ATR accessory (Golden Gate, Graseby Specac Ltd., Kent, England). Following established procedures,^40^ “each spectrum was collected after 16 scans between 4000 and 600 cm^–1^ at intervals of 1 cm^–1^ with a resolution of 4 cm^–1^”. The FTIR spectra were fitted by an automatic baseline correction using OMNIC 4.0 software. The crystallinity fraction (CI) of cellulose was calculated following the method of Nelson and O’Connor,^50^ as previously described.^51,52^

#### 2.4.7 Density

To assess the apparent density, square film samples of 2 cm sides were equilibrated under controlled conditions (23 °C, 50% relative humidity) for 1 day. The ratio of mass to volume was then determined for all AX-based films.

#### 2.4.8 Tensile Properties

The tensile strength, strain, and modulus of the films were determined as previously described^53^ using an “Instron Universal Testing Machine model 5944 (Instron Engineering Corporation, MA) equipped with pneumatic jaws and a 250 N load cell”. Prior to mechanical testing, film samples were conditioned at 23 °C and a relative humidity of 50%. According to the ASTM D882-09 standard, five samples measuring 5 mm by 50 mm were evaluated using a starting clamp distance of 20 mm and a grip strain rate of 2 mm min^–1^.

#### 2.4.9 Thermogravimetric Analysis

The thermal behavior of the biomass fractions (RB, RB-A, RB-B, RB-CNC) and films (R-AX-F, R-AX/CNC5, R-AX/CNC10, and R-AX/CNC20) was evaluated using a Mettler Toledo TGA/DSC 1 STAR module (Columbus, OH), following previously published methodologies.^49^ Approximately 4–5 mg of each sample “was heated from 25 °C to 800 °C at a rate of 10 °C/min in inert atmosphere (of 50 mL min^–1^ N_2_ flow)”. The thermal parameters of each mass loss (the temperature of maximum weight loss (*T*_max_) and the initial decomposition temperature (*T*_onset_)) were determined as indicated in previous publications.^51,52^ All measurements were performed in triplicate.

#### 2.4.10 Transparency and Haze

The optical characteristics of all the processed films were analyzed as previously^53,54^ “by using a Shimadzu UV-250 spectrophotometer (Shimadzu Corporation, Japan) equipped with an integrating sphere”. Transparency was assessed by recording UV–vis spectra over the wavelength of 220–800 nm range, while haze measurements were evaluated from 400 to 800 nm wavelength range.

#### 2.4.11 Water Vapor Transmission Rate and Permeability

The water vapor barrier performance of the films was measured following the same procedure previously published.^53^ According to the norm NF H 00-03022 “the films at 23 °C and 50% RH was measured by duplicate using CaCl_2_ as the desiccating agent”. “The films were placed in a cup with a cross-sectional area of 5 cm^2^ and weighted every hour”. Water vapor transmission rate (WVTR) (g m^–2^ day^–1^) was calculated as the ratio of the cup’s mass increase over time (g day^–1^) to the exposed film area (m^2^). WVP, expressed in g mm m^–2^ day^–1^ Pa^–1^, was determined by first multiplying the WVTR by the average film thickness. This was then divided by the water vapor pressure gradient across the films.

#### 2.4.12 Statistical Analysis

To evaluate variations between the mean values of film properties, a one-way ANOVA was performed. Posthoc comparisons were conducted using Tukey’s test, and statistically significance was established at a confidence level of *p* < 0.05.

## 3 Results and Discussion

### 3.1 Isolation of Cellulose Nanocrystals from Rye Bran

The recovery yields and “chemical compositions (carbohydrate, Klason lignin, ash, and extractive content)” of rye bran (RB) and its isolated fractions (RB-A, RB-B and RB-CNC) were analyzed after each processing treatment (Table 1). Total carbohydrate composition was quantified by measuring neutral sugars, as well as uronic acids. As reported in previous studies,^8,40^ glucans in rye bran (RB) were primarily attributed to cellulose, but also to residual starch and β-glucans. However, starch and β-glucans were removed after alkaline treatment, therefore the glucose content in the isolated fractions can be directly assigned to cellulose. On the other hand, the hemicellulose/pectin content was determined based on the percentage of residual sugars, primarily consisting of AX with minor arabinogalactan and galactomannan portions.

**Table 1:** Yield and Chemical Composition of Rye Bran and its Fractions Obtained After Different Treatments During the CNC Isolation Process

	RB	RB-A	RB-B	RB-CNC
yields (% DW)^a^	100	38.5 ± 7.1	63.0 ± 5.0	25.5 ± 3.0
carbohydrate content (g 100 g^–1^)^b^	58.8 ± 4.0	72.1 ± 6.0	85.8 ± 1.0	92.6 ± 3.7
arabinose (%)^c^	12.1 ± 0.4	6.2 ± 1.6	4.2 ± 0.1	<0.1
galactose (%)^c^	1.0 ± 0.1	2.8 ± 0.4	0.7 ± 0.05	<0.1
glucose (%)^c^	66.0 ± 0.2	75.9 ± 3.9	83.9 ± 0.4	97.8 ± 1.0
xylose (%)^c^	20.6 ± 0.5	11.9 ± 0.1	9.0 ± 0.2	<0.1
mannose (%)^c^	0.3 ± 0.08	3.0 ± 0.3	2.6 ± 0.0	1.8 ± 0.7
galacturonic acid (%)^c^	0.1 ± 0.01	<0.1	<0.1	<0.1
glucuronic acid (%)^c^	<0.1	<0.1	<0.1	<0.1
glucans^d^–cellulose (g 100 g^–1^)^e^	38.8 ± 1.3^d^	54.8 ± 2.4^e^	72.0 ± 0.3^e^	90.6 ± 2.8^e^
hemicellulose/pectin (g 100 g^–1^)^f^	20.0 ± 2.7	17.4 ± 1.6	13.8 ± 0.7	2.0 ± 0.9
Klason lignin (%)^g^	10.5 ± 1.6	8.4 ± 1.0	2.8 ± 0.6	N/A^j^
ash (%)^h^	7.0 ± 0.5	7.8 ± 1.2	3.8 ± 0.2	3.0 ± 1.0
extractives (%)^i^	7.7 ± 1.0	N/A^j^	N/A^j^	N/A^j^

a Gravimetric yields calculated as % of the dry weight obtained after each individual treatment.

b Total carbohydrate content quantified based on HPAEC-PAD analysis of the hydrolyzed monomers.

c Relative abundance (wt %) of monosaccharides in the total carbohydrate fraction.

d This value largely attributed to the presence of cellulose, but may also include residual starch after hydrolytic degradation, along with minor amounts of mixed-linkage β-glucans.

e Cellulose determined as the glucose detected.

f Hemicellulose/pectin amount calculated from the percentages of arabinose + galactose + xylose + mannose + galacturonic acid.

g Lignin content determined by Tappi T222 om-06 test method.

h Ash content evaluated using TGA.

i Water/ethanol extractives.

j N/A = not applicable.

The chemical composition of RB was within the range reported in the literature,^7,8^ containing approximately 60% carbohydrates (including 40% glucans and 20% hemicellulose/pectin), 10.5% Klason lignin, 7% ash, and 7.7% extractives. The hemicellulose/pectin fraction was primarily composed of arabinoxylan, with arabinose and xylose accounting for 12.1% and 20.6% of the total carbohydrates, respectively. This corresponds to an arabinoxylan content of 19.2 g per 100 g of RB, and to an Ara/Xyl ratio of 0.58. These findings align with previously reported AX content values for Nordic rye brans^55^ and support earlier studies suggesting that bran AX is less branched than AX in the endosperm, where the Ara/Xyl ratio typically ranges from 1 to 1.2.^55–57^

During the alkali treatment, more than 60% of the initial RB mass was lost, resulting in a biomass recovery of only 40%. This yield is among the lowest reported in the literature for residual biomasses subjected to similar alkaline treatments.^8,40–42^ This significant mass reduction can be attributed not only to the removal of extractives and lignin but also to the elimination of the relatively high protein and starch content present in RB. Although these components were not individually quantified in the RB chemical composition analysis, they may account for up to 15% of the original material, as the sum of identified compounds constitutes only 85% of the initial biomass. Despite this low recovery, the alkalinized sample (RB-A) exhibited a significant increase in carbohydrate content, achieving a cellulose purity of 51%, similar to values reported for alkalinized rice husks^40^ and other lignocellulosic forest residues.^42^

Following the alkali step, the RB-A sample was submitted to a bleaching process, recovering almost 63% of the treated biomass. This yield aligns with previously reported recovery values from rice husks,^40^ pine cones,^41^ spruce bark,^48^ and woody chips,^42^ which were submitted to bleaching after an alkaline treatment. The bleaching treatment significantly reduced lignin and ash content, further enriching the carbohydrate concentration of the isolated fraction. The resulting bleached fraction (RB-B) appeared as a white, fibrous material (Figure 1A), with a cellulose content over 84% of the total carbohydrate fraction, highlighting its potential as a feedstock for CNC production through acid hydrolysis.

**Figure 1:**
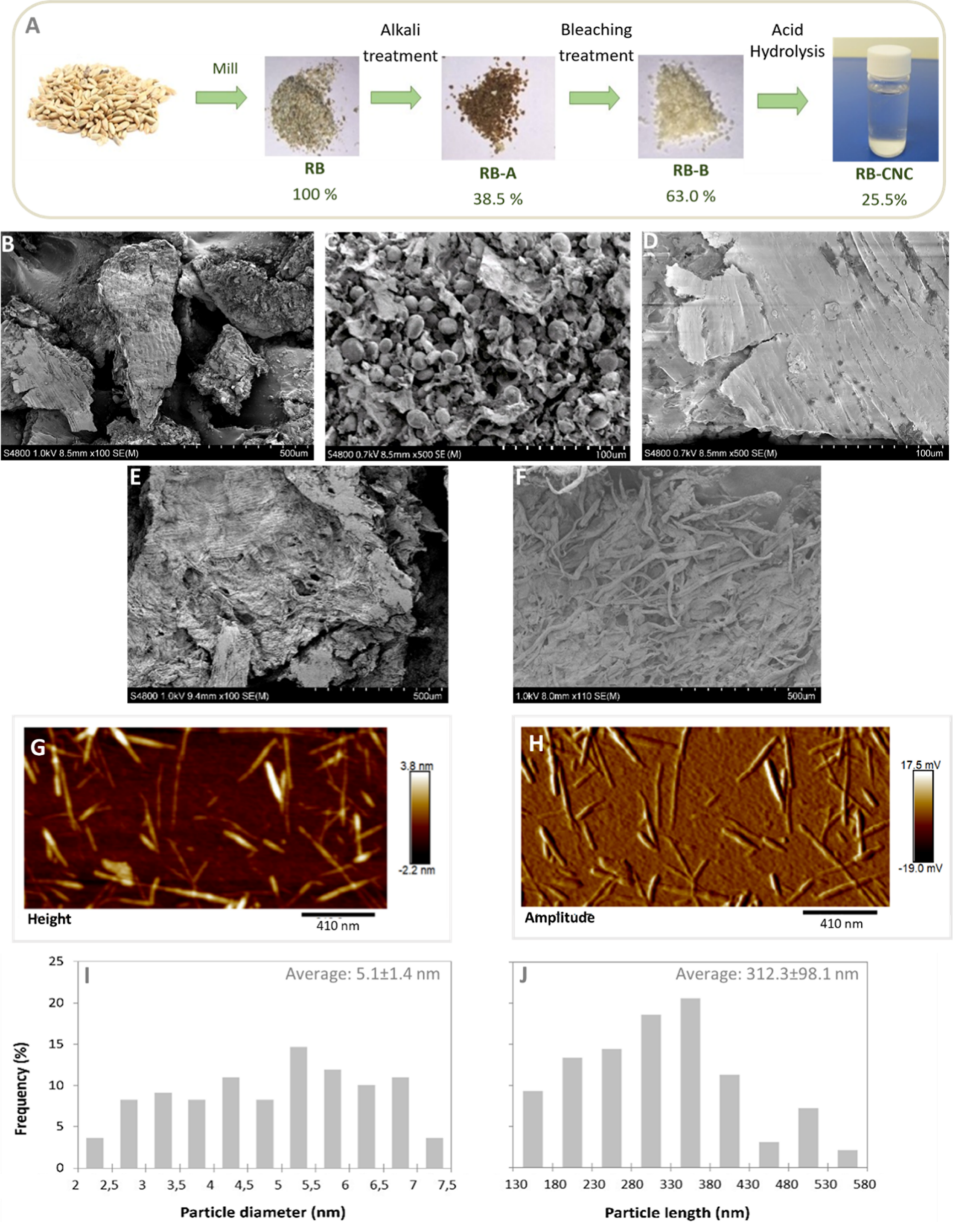
Evolution of the isolation of cellulose nanocrystals from rye bran (RB-CNC): (A) schematic representation of the RB-CNC isolation process, showing the gravimetric yields at each processing stage, determined relative to the dry weight from the preceding step. (B–F) SEM of: (B) untreated milled rye bran (RB), (C) detailed morphology of the inner epidermal surface of RB, (D) detailed morphology of the outer epidermal surface of RB, (E) rye bran after alkali treatment (RB-A), and (F) alkalinized rye bran after bleaching (RB-B). (G–J) Morphological characterization of RB-CNC: AFM images of RB-CNC in (G) height and (H) amplitude mode, and (I) particle diameter and (J) length distributions, calculated from image analyses of 100 individual RB-CNC nanoparticles.

The CNC recovery yield from RB-B was 25.5%, consistent with previously reported values for CNC isolated from rice bran^23^ and rice straw.^26^ This yield exceeds those reported for CNC derived from barley straw and husks (<4%),^20^ as well as rice husks (14%).^40^ However, it remains lower than those reported for other forest residual biomasses^41,42,48,58^ and purified cellulose sources (e.g., bleached kraft pulp and microcrystalline cellulose),^26^ which can reach up to 33% under similar hydrolytic isolation conditions. These findings highlight the inherent limitations in CNC yield when using agro-industrial lignocellulosic feedstocks, particularly cereal-based biomass, which typically contains less cellulose than woody biomass.^26^ An overall yield of 6.2% was achieved for RB-CNC, based on the starting mass of rye bran. This value aligns with previously reported yields for CNC isolated from other agricultural residues^47^ and even surpasses those reported from certain cereal biomasses, including rice husk (4%)^40^ and wheat straw (4.83%).^26^ Given this modest overall yield, CNC production from rye bran alone may not represent the most efficient route for biomass valorization. However, it can serve as a valuable step within a multiproduct biorefinery framework, where CNC extraction may be integrated into a broader fractionation strategy. In such an approach, multiple components of rye bran may be sequentially recovered and utilized. For example, AX can be extracted during upstream processing steps (e.g., alkaline treatment), as demonstrated in previous studies by Svärd et al.^47^ and Requena et al.^40^ This integrated strategy may enhance overall resource efficiency and support a more sustainable utilization of rye bran.

During the hydrolytic treatment hemicelluloses/pectins and the amorphous regions of cellulose were hydrolyzed and solubilized, yielding a highly purified RB-CNC aqueous suspension (Figure 1A) with a cellulose content of nearly 90%. This purity is comparable to CNC obtained from rice husks^40^ and higher than those derived from forest residues^41,42,48^ under similar conditions. The RB-CNC suspension exhibited a zeta potential (ζ) value of −35.8 ± 0.4 mV, indicating high colloidal stability. Absolute ζ-values exceeding 30 mV are typically associated with highly stable and uniform CNC suspensions, with well-dispersed nanoparticles resistant to aggregation over time.^59^ This strong colloidal stability of RB-CNC can be attributed to the esterification of hydroxyl groups by sulfate ions, which introduced surface charges and generated electrostatic repulsion between nanoparticles.^60^ This high ζ-value, therefore, indicates that RB-CNC suspension can be effectively incorporated into waterborne formulations for film processing, ensuring good dispersibility and preventing nanoparticle agglomeration, which may alter the properties of the resulting materials and potentially reduce mechanical strength.^61^

To investigate the structural changes occurring during the conversion of RB from macro- to nanoscale, surface features were visualized after sequential alkali and bleaching treatments (Figure 1B–F), while AFM evaluated the morphology and size distribution of RB-CNC (Figure 1G–J). SEM analysis of untreated RB (Figure 1B and its magnification, Figure 1C) revealed starch granules with a bimodal size distribution, with larger lenticular-shaped and smaller spherical-shaped granules ranging from 6 to 25 μm.^62^ The presence of starch in the RB samples highlights the difficulty of separating the starchy endosperm from the pericarp and aleurone layers during rye milling,^5^ and supports the previously hypothesized high starch content in the RB chemical composition. In contrast, Figure 1E shows the effective removal of starch granules and the appearance of a more swollen and structurally deteriorated cell wall on the RB-A surfaces, compared to the untreated ones (Figure 1D). Further bleaching (Figure 1F) resulted in a partial separation of fiber bundles in the RB-B samples, confirming the presence of amorphous cell wall components (primarily hemicelluloses (17%) and lignin (3%), identified by chemical analysis), which act as structural binders holding the fiber bundles together. AFM images of the produced RB-CNC in height and amplitude mode are displayed in Figure 1G,H respectively, revealing nanoparticles with the characteristic rod-like shape of CNC together with some aggregates, as a result of intermolecular forces between crystals.^48^ The size profiles, including their lengths and diameters, were measured and represented in histograms (Figure 1I,J). The RB-CNC size distribution was within the typical range expected for CNC isolated from plant biomass (diameter between 2 and 20 nm and length between 100 and 600 nm).^63^ Specifically, the RB-CNC displayed a diameter ranging from 2 to 7.5 nm and a length between 130 and 460 nm. This size polydispersity is slightly narrower than that reported for CNC isolated from rice husk, despite the latter being produced under a longer hydrolytic time. Figure 1I,J also shows the diameter (*d*) and length (*l*) of the isolated RB-CNC, expressed as mean values. A similar average diameter (∼5 nm) has been reported for rice husk-derived nanocrystals;^40^ however, RB-CNC displayed a greater average length (312.3 nm)^40^ similar to that reported for CNC derived from barley husk.^64^ On the other hand, CNC derived from forest typically displayed lower average lengths, not higher than 200 nm.^42,48^ This finding corroborates previous studies by Espino et al.,^64^ which demonstrated that longer CNC can be produced from cereal brans rather than forest biomass, leading to a higher aspect ratio and greater potential for reinforcement in renewable nanocomposites at low loads.^64^ The combination of the high length and small diameter of RB-CNC resulted in one of the highest average aspect ratios (l/*d* = 61) reported for hydrolytically produced CNC.^65^ As a result, RB-CNC may offer even greater reinforcing potential than CNC derived from other agricultural residues and byproducts (such as coconut husks,^66^ rice husks,^40^ barley husks,^64^ mengkuang leaves^67^ and rapeseed straw^47^), which generally exhibit lower aspect ratios (<47).

The starting biomass and the resulting fractions produced through each chemical treatment were also physicochemically characterized using FTIR and TGA to gain further insight into the CNC isolation process. As shown in the FTIR spectra (Figure 2A), a progressive increase in cellulose content was observed across the alkaline, bleaching, and hydrolytic treatments. The broad absorption band observed around 3297 cm^–1^, associated with the OH stretching of aliphatic hydroxyl groups in carbohydrates, became narrower, indicating the progressive elimination of amorphous regions and an enhancement in crystalline structure as the CNC isolation process advanced.^42^ In the RB-CNC, two overlapping OH stretching peaks near 3336 cm^–1^, and 3278 cm^–1^ were identified, attributed to intramolecular (3OH···O5) and intermolecular (6OH···O3) hydrogen bonding, respectively, as previously reported in CNC derived from other residual biomass.^42^ The peaks centered around 2930 and 2850 cm^–1^, associated with the antisymmetric and symmetric CH2 stretching vibrations of noncellulosic polysaccharides,^68^ gradually diminished after the alkaline treatment, resulting in a single peak at 2908 cm^–1^, corresponding to the CH stretching vibrations in cellulose. The band observed at 1720 cm^–1^, characteristic of C=O vibrations, typically associated with carboxylic acids from hemicelluloses side-chains and/or lignin-carbohydrate-complex bonds,^69^ disappeared after the alkali treatment.^70^ However, despite the absence of this peak, chemical analysis confirmed that the RB-A sample still contained significant amounts of hemicelluloses. These findings suggested that alkalinization promotes hemicelluloses debranching, leading to their autohydrolysis, and were consistent with previous results in alkaline-treated forest residues.^71^ Furthermore, the intensities of lignin-related peaks at 1510 cm^–1^, and 1250 cm^–1^ ^42^ (assigned to the aromatic ring vibration (C=C) and the C–O bond stretching associated with lignin, respectively) were significantly reduced after bleaching, corroborating the strong delignification indicated at this step in the chemical analysis.

**Figure 2:**
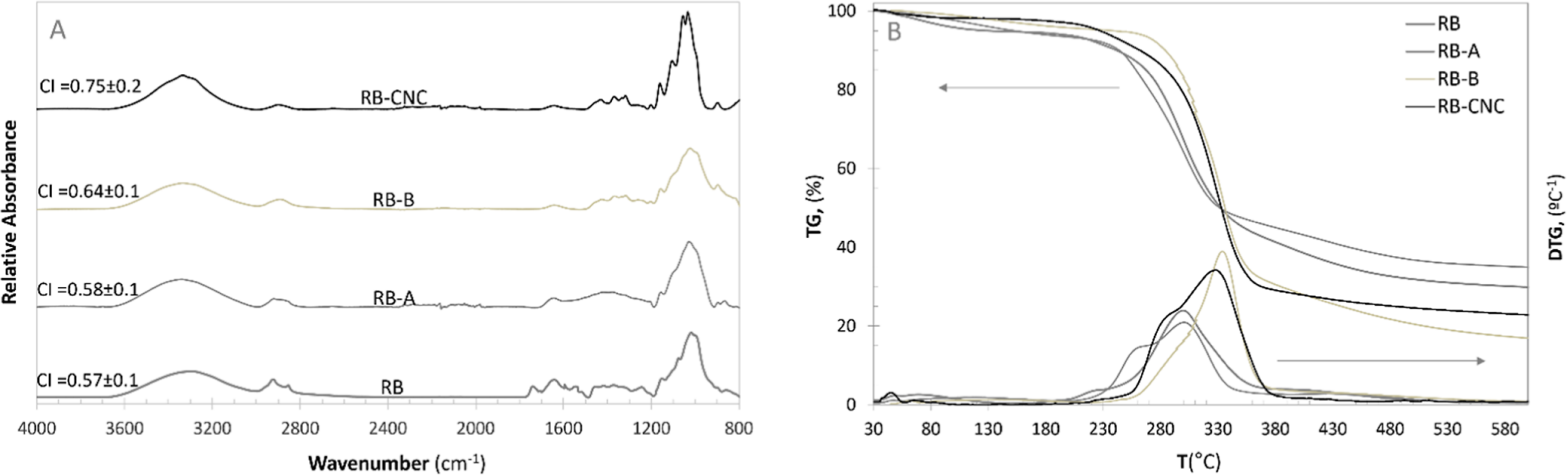
Chemical structure and thermal behavior of rye bran (RB) and its isolated fractions obtained during the alkaline (RB-A), bleaching (RB-B) and hydrolysis (RB-CNC) treatments: (A) FTIR spectra; and (B) thermogravimetric (TG) and derivative thermogravimetric (DTG) curves.

The cellulose crystallinity was estimated by FTIR using an empirical parameter called the crystallinity fraction (CI), originally defined by Nelson and O’Connor,^68^ based on the intensity ratio of the absorption peaks at 1371 and 2900 cm^–1^. A slight increase in CI from 0.57 in RB to 0.64 in RB-B indicated a minimal removal of amorphous cellulose during alkaline and bleaching treatments. In contrast, previous studies reported a more significant increase in CI (from 0.42 to 0.83)^53^ after the bleaching, suggesting that in those cases, the chemical treatments applied prior to hydrolysis led to the elimination of amorphous regions, including portions of amorphous cellulose. In the present study, the comparatively modest increase in CI suggested that the alkaline and bleaching treatments preserved the cellulose structure with minimal degradation, including limited removal of amorphous cellulose. As a result, during subsequent acid hydrolysis, sulfuric acid may predominantly act on the remaining amorphous regions without significantly eroding the crystalline domains, an effect that becomes more pronounced under harsher hydrolysis conditions.^23^ This preservation of the cellulose structure prior to hydrolysis may explain the longer RB-CNC found in this study.

Figure 2B displays the TG and DTG curves of all the studied samples showing two main mass-loss regions. The first region of the RB samples, occurring below 150 °C, showed a minor mass loss of 5.6 ± 0.3% with a peak centered at 70.1 ± 1.2 °C, which was attributed to moisture evaporation. The second region of the RB samples (from 155 to 550 °C) with a mass loss of 66.2 ± 2.0%, displayed a multistage thermal degradation characterized by a prominent peak near 300.6 ± 0.2 °C attributed to cellulose degradation, an overlapping peak at 220.9 ± 0.9 °C related to noncellulosic polysaccharides, and a high-temperature tail (above 360 °C), associated with lignin decomposition.^51^ The progressive removal of amorphous components during alkali and bleaching treatments resulted in a narrower peak at higher decomposition temperatures (337.5 ± 0.6 °C) corresponding to cellulose decomposition overlapped with a minor peak at lower temperatures related to the hemicelluloses still present in the RB-B chemical composition. The pyrolysis of RB-CNC occurred through two overlapping processes, consistent with the typical decomposition pattern of CNC,^26^ initial thermal degradation of the more accessible sulfated amorphous region (centered at 286.1 ± 0.6 °C), followed by the breakdown of the less accessible crystalline regions (centered at 332.3 ± 0.6 °C).^64^

The onset temperature (*T*_onset_) determines the “thermal stability related to the initial decomposition temperature” at which the thermal degradation begins.^72^ The *T*_onset_ of RB was 198.4 ± 2.6 °C, indicating lower thermal stability than that of rice husk,^40^ probably due to their differences in their chemical composition. After the alkaline and bleaching treatments, the *T*_onset_ increased to 259.2 ± 0.1 °C in RB-B. This significant increase of ∼60 °C in the *T*_onset_ was attributed to the elimination of less thermally stable amorphous components, leading to enhanced crystallinity and a higher proportion of intermolecular hydrogen bonding domains.^58^ As expected, sulfate groups bound to the glucose units catalyzed the dehydration process of cellulose, decreasing the thermal stability to 230.0 ± 4.2 °C in the RB-CNC samples. However, this value was still higher than those previously reported for CNC derived from other cereal brans, such as barley husks (217 °C) and rice husks (207 °C) and other residual biomasses (such as, pine needles (211 °C),^42^ spruce bark (190 °C),^48^ pine cone (150 °C)).^41^ These findings highlight the potential of rye bran derived CNC for being used in thermally processed composites, as their *T*_onset_ exceeded typical processing thresholds, demonstrating exceptional thermal stability among CNC.

### 3.2 Cellulose Nanocrystals from Rye Bran as Reinforcement in Arabinoxylan-Based Composite Films

Composite formulations derived 100% from rye biomass were developed using commercial rye arabinoxylan (R-AX) as polymeric matrix and three different concentrations (5%, 10%, and 20%) of isolated rye bran cellulose nanocrystals (RB-CNC) as reinforcing agents. Both the neat R-AX and the composite formulations exhibited excellent film-forming properties, producing self-standing, uniform, and transparent films coded as follows: R-AX-F, R-AX/CNC5, R-AX/CNC10 and R-AX/CNC20 (Figure 3A). Surface topography of the films was analyzed by AFM (Figure 3B). In all the composite formulations, the RB-CNC appeared to be fully embedded and homogenously distributed within the R-AX matrix. The RB-CNC exhibited random orientation across all samples; however, in the R-AX/CNC20 film, the RB-CNC appeared more densely packed and interconnected.^49^

**Figure 3:**
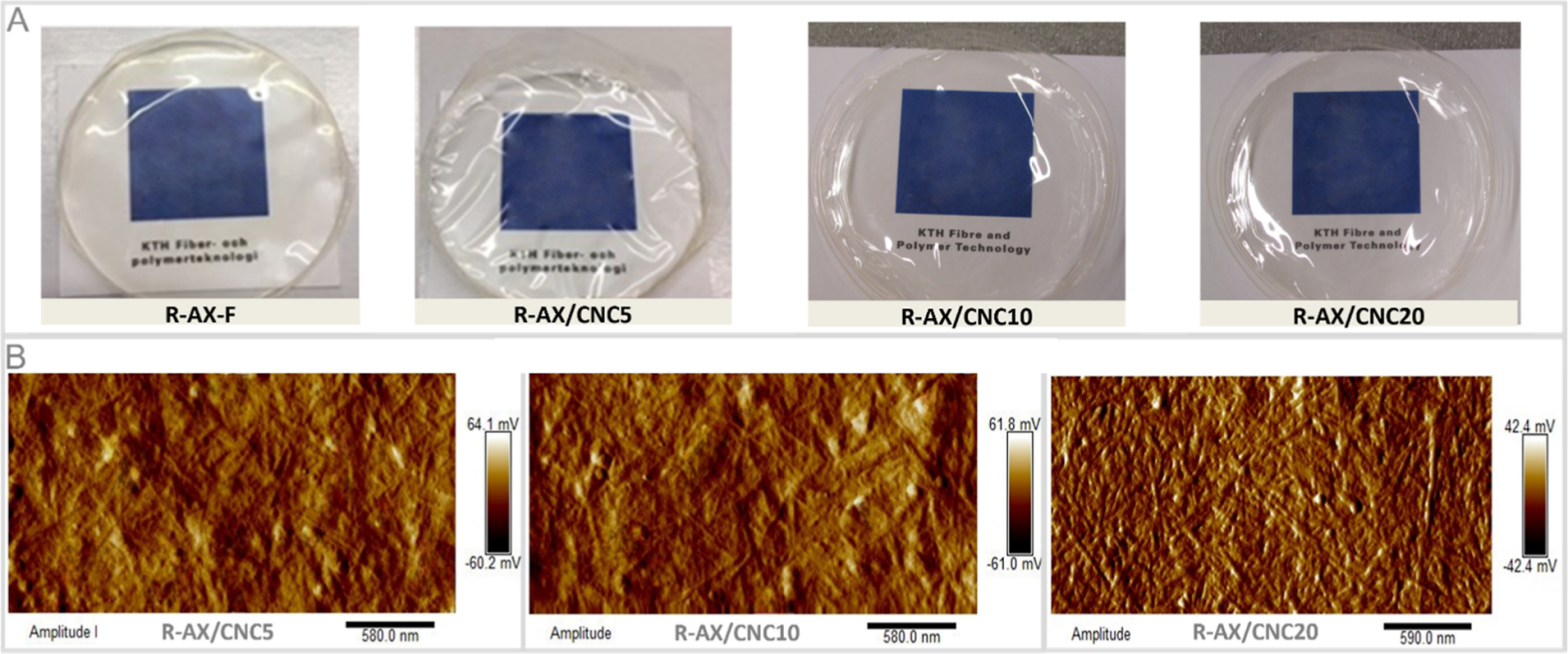
(A) Free-standing films with different formulations. (B) AFM amplitude-mode images of RB-CNC reinforced nanocomposite films.

The resulting films had a grammage of approximately 40 g m^–2^, which is a typical value for thin to medium weight transparent food-packaging films. This grammage is also comparable to that used in various paper- and fiber-based packaging applications, including waterborne dispersion coatings for paper bags and polymeric layers on coated packaging boards.^73^ The average thickness of the films varied from 18 to 23 μm, with differences depending on the RB-CNC load. As shown in Table 2, the thickness of R-AX-F increased with the addition of 5% RB-CNC; however, the average thickness of R-AX/CNC10 and R-AX/CNC20 composite films was lower than that of the R-AX/CNC5, showing a general decreasing trend when increasing RB-CNC content. This reduction in the thickness resulted in a higher density, suggesting strong interfacial interactions between the components and enhanced molecular packing within the composite structure at higher reinforcement loads.^39,54^ Similar density values were previously reported for edible composite films containing wheat AX and cellulose nanofibrils (CNF).^34^

**Table 2:** Influence of RB-CNC Content on the Average Thickness, Apparent Density, Water Vapor Transmission Rate and Permeability of the Reinforced Composite Films

	thickness [μm]	density [g cm^–3^]	WVTR [g m^–2^ day^–1^]	WVP [g mm m^–2^ day^–1^ kPa^–1^]
R-AX-F	20.8 ± 0.4^a^	1.60 ± 0.04^a^	132.3 ± 10.5^a^	1.96 ± 0.15^a^
R-AX/CNC5	23.0 ± 1.5^b^	1.73 ± 0.12^a^	132.4 ± 8.7^a^	2.17 ± 0.24^a^
R-AX/CNC10	19.3 ± 1.0^a^ ^,^ ^c^	2.03 ± 0.12^b^	148.0 ± 9.9^a^	2.03 ± 0.08^a^
R-AX/CNC20	18.3 ± 1.2^c^	2.14 ± 0.16^b^	137.2 ± 8.6^a^	1.80 ± 0.16^a^

a Mean values and standard deviations marked with different letters have statistical differences (*p* < 0.05).

b Thickness and density—average of 8–10 replicates and Tukey’s post hoc test.

c WVTR and WVP properties—average of 2–4 replicates without significant difference in mean values between the groups.

The reinforcing potential of RB-CNC in R-AX-based composite films was systematically assessed by evaluating key packaging-relevant properties, including water vapor permeability (WVP), mechanical performance, thermal stability, and optical characteristics (i.e., transparency and haze). WVP is a key property to be evaluated in the design of biobased materials for food packaging, as it plays a critical role in moisture-induced spoilage caused by moisture migration between the food and its surrounding atmosphere.^74^ Table 2 summarizes the WVTR and the corresponding WVP values for all formulated films. Hemicellulose-based films, particularly those made from unmodified AX, are known for their high moisture sensitivity, often resulting in elevated WVTR and WVP values.^75^ Unplasticized AX films derived from wheat^32,76^ and oat spelt^30^ have shown some of the lowest reported WVTR values (90 and 186 g m^–2^ day^–1^, respectively) for films with an average thickness of approximately 45 μm. In this study, R-AX-F films exhibited a WVTR value of 132 g m^–2^ day^–1^, falling within the previously reported range for unmodified AX films. However, since the R-AX-F films were approximately half as thick, their corresponding WVP value of 2.27 × 10^–11^ g m^–1^ s^–1^ Pa^–1^ represents the lowest reported value to date for unplasticized AX films.^77^ This barrier performance is comparable or even superior to that reported for chemically and physically modified AX films. For instance, 10% sorbitol-plasticized corn hull AX films,^78^ with a thickness of 32 μm, displayed WVTR values around 118 g m^–2^ day^–1^ and WVTP values of around 2.6 × 10^–11^ g m^–1^ s^–1^ Pa^–1^. Similarly, sorbitol-plasticized AX films from oat spelt^35^ (25–50 μm) and acetylated wheat AX films^32^ (45 μm) exhibited WVP values of around 1.9 g mm m^–2^ day^–1^ kPa^–1^ and they were noted as some of the lowest values previously reported for modified AX films.^77^ These results highlight the potential of using R-AX films for packaging applications, as they offer equivalent or improved water barrier properties without relying on plasticizers or cross-linkers.

The incorporation of CNC into polysaccharide-based films is commonly associated with a reduction in WVTR,^65^ often accompanied by an increase in film thickness. This enhancement in barrier performance is typically attributed to the ability of CNC to enhance the tortuosity of the diffusion pathway within the polymeric matrix, slowing down water vapor diffusion and improving moisture resistance.^74^ However, in the present study, increasing RB-CNC content in the film formulations did not lead to a significant reduction in WVP values, instead the WVP remained comparable to that of neat R-AX films while increasing their density (Table 2). This result suggested that the progressive formation of tighter intermolecular networks with increasing RB-CNC content did not significantly elongate the diffusion pathway, compromising the expected barrier enhancement. A similar phenomenon was previously reported in nanocomposite systems undergoing cross-linking reactions, where excessive network compaction together with the addition of nanocelluloses did not show a clear reduction of WVP.^77^

Compared to other polysaccharide-derived films, the WVP values obtained in this study were comparable to those of methylcellulose (MC) films with paraffin oil (2.4 × 10^–11^ g m^–1^ s^–1^ Pa^–1^) and notably better than those of neat MC films (8.4–12 × 10^–11^ g m^–1^ s^–1^ Pa^–1^), chitosan (CH) and CH-MC composite films (3.7–15.27 × 10^–11^ g m^–1^ s^–1^ Pa^–1^), corn starch derived films with CH (4.46 × 10^–11^ g m^–1^ s^–1^ Pa^–1^), amylomaize starch with sorbitol and sunflower oil (9.7 × 10^–11^ g m^–1^ s^–1^ Pa^–1^), and cellophane (8.4 × 10^–11^ g m^–1^ s^–1^ Pa^–1^).^79^ Therefore, it can be concluded that WVP performance of all the designed film was comparable or superior to that of other commercial biopolymers such as cellophane and corn starch films used in food packaging applications.

Assessing the tensile properties of films is crucial in biobased packaging, as it determines their mechanical strength and flexibility, ensuring they can withstand the stresses of handling, storage, and transportation demands.^6^ Table 3 summarizes the tensile properties of neat rye arabinoxylan films (R-AX-F) and their RB-CNC-reinforced composites. The obtained mechanical properties of R-AX-F films were comparable to previously reported data for R-AX films, exhibiting exceptional stiffness (2.1 GPa) together with a significant tensile strength (62 MPa), and a moderate flexibility (10.4%).^36,39^ These mechanical properties are among the best values reported for unmodified AX films, outperforming films derived from other cereal sources such as wheat, oat and barley, regardless of the plant part used for AX extraction (e.g., bran, straw/stalk, endosperm).^27,30^ The incorporation of RB-CNC into R-AX formulations significantly enhanced their strength and stiffness while retaining the flexibility across all formulations, highlighting the strong reinforcing capability of RB-CNC in R-AX based composites.^39^ Specifically, the addition of 5% RB-CNC led to significant increases of 77% in Young’s modulus and 37% in tensile strength compared to R-AX-F. This reinforcing effect of RB-CNC was superior to that observed when 5% bacterial nanocellulose was incorporated into similar R-AX films,^36^ which only achieved improvements of 8% and 17% in Young’s modulus and tensile strength, respectively. These results suggested that RB-CNC offers superior reinforcing potential compared to bacterial cellulose, likely due to its combined effects of higher surface charge and exceptional aspect ratio, which promote the formation of a mechanically percolated network through hydrogen bonding interactions between well-dispersed individual nanocrystals.^38^ As the RB-CNC content increased to 10%, further improvements in all evaluated tensile properties were achieved, with Young’s modulus increasing by 81%, elongation at break by 49%, and tensile strength by 98%, compared to R-AX-F. However, this increasing trend became less pronounced at 20% RB-CNC loading, resulting in only a marginal improvement in Young’s modulus and a significant reduction in tensile strength and elongation when compared with R-AX/CNC10. This may be related to reaching the percolation threshold, where further increases in RB-CNC content led to greater nanoparticle interactions, leading to the formation of a stiffer network characterized by stress-concentrating regions, making the films more brittle and less tough.^64^

**Table 3:** Effect of RB-CNC Content on the Physical Performance of Rye AX-Based Films; Average Thickness, Mechanical and Thermal Properties of Neat R-AX-F and its Reinforced Composites

films	mechanical properties			thermogravimetric properties					
	Young’s modulus [GPa]	elongation at break [%]	tensile strength [MPa]	region I (25–155 °C)		region II (160–550 °C)			residue [%]
				mass loss [%]	*T*_max_ [°C]	*T*_onset_ [°C]	mass loss [%]	*T*_max_ [°C]	
R-AX-F	2.1 ± 0.3^a^	10.4 ± 1.1^a^	62.5 ± 3.3^a^	5.6 ± 0.2	84.9 ± 1.5^a^	251.8 ± 2.0^a^	62.2 ± 0.6^a^	278.2 ± 1.2^a^	29.3 ± 0.2^a^
R-AX/CNC5	3.7 ± 0.8^b^	10.9 ± 1.8^a^	88.6 ± 5.7^b^	5.7 ± 0.2	72.8 ± 0.3^b^	263.5 ± 1.4^b^	72.0 ± 1.4^b^	290.2 ± 0.3^b^	21.8 ± 0.2^b^
R-AX/CNC10	4.2 ± 1.1^b^	16.9 ± 1.5^b^	111.9 ± 1.8^c^	5.7 ± 0.1	73.5 ± 0.0^b^	261.6 ± 1.0^b^	72.0 ± 0.5^b^	287.2 ± 2.4^b^	21.9 ± 0.2^b^
R-AX/CNC20	4.4 ± 0.2^b^	12.7 ± 2.9^a^ ^,^ ^b^	86.1 ± 3.2^b^	5.9 ± 0.2	74.2 ± 0.7^b^	263.5 ± 0.6^b^	75.0 ± 1.2^b^	289.5 ± 2.4^b^	18.3 ± 1.2^c^

a Mean values and standard deviations marked with letters have statistical differences (*p* < 0.05).

b Thickness-average of 8–10 replicates and Tukey’s post hoc test.

c Mechanical properties—average of 4–5 replicates and Tukey’s post hoc test; thermogravimetric properties—average 2 replicates and Tukey’s post hoc test.

An estimation of the percolation threshold of the RB-CNC in the R-AX matrix was carried out based on their rod-like geometry and random orientation in the composite films. Using experimentally determined average dimensions (length = 312.3 ± 98.1 nm; diameter = 5.1 ± 1.4 nm), and theoretical models for nanocomposite films with randomly dispersed rod-shaped fillers, the percolation threshold is expected to occur approximately between 5.3–17.7 wt %, with a central estimate near 9.6 wt %. This range aligns well with the observed mechanical property enhancements, particularly the sharp improvements at 10 wt % RB-CNC, suggesting the formation of a percolated nanocrystal network that reinforces the film structure.

Despite this, R-AX/CNC20 films still displayed better mechanical properties than R-AX/CNC5 and R-AX-F films, demonstrating the persistence of a percolated structure, albeit one with limited incremental benefit beyond the optimal threshold. This behavior illustrates the nonlinear relationship between CNC content and mechanical performance in nanocomposites, where optimal reinforcement is achieved near the percolation threshold, while excessive loading may lead to diminished or plateauing returns due to aggregation and stress localization.^26^

When compared the obtained mechanical properties with those from synthetic packaging films, it can be observed that Young’s modulus, tensile strength and elongation at break of all the developed composite films were in the range of those for cellophane with approximate values of 3 GPa, 85.8 MPa and 14.4%, respectively.^78,79^

Investigating the thermal behavior of CNC-reinforced polysaccharide-based composites, which typically exhibit limited thermal stability due to the sulfate groups bounded to the CNC, is crucial for assessing their suitability for packaging applications and determining their thermal processability. The TG curves of all the formulated films (Figure 4A) displayed a main thermal decomposition process occurring between 155 and 550 °C, attributed to the degradation of the R-AX chains,^80^ with mass loss exceeding 62%. This mass loss progressively increased with higher RB-CNC content and consequently the residual mass was reduced (Table 3). In Figure 4A, the DTG curve of R-AX-F displayed a narrow and well-defined peak around 278 °C, with an onset temperature of 252 °C. As RB-CNC content increased, an additional overlapping peak around 350 °C emerged due to the degradation of the RB-CNC, which took place between 230 °C and 380 °C. The incorporation of RB-CNC into AX-based formulations resulted in films with higher thermal stability, regardless of the reinforcement content. This improvement in thermal stability is typically attributed “to strong interfacial interactions” (particularly hydrogen bonding) between composite components.^49^ These interactions improve component compatibility and restrict polymer chain mobility, thus delaying thermal degradation.^53,81^ R-AX/CNC20 films exhibited an onset value of 262 °C that was significantly higher than those values reported for other CNC-reinforced polysaccharides films,^49,81,82^ similar to those exhibited for bioplastics, such as PLA^83^ and cellophane.^79^

**Figure 4:**
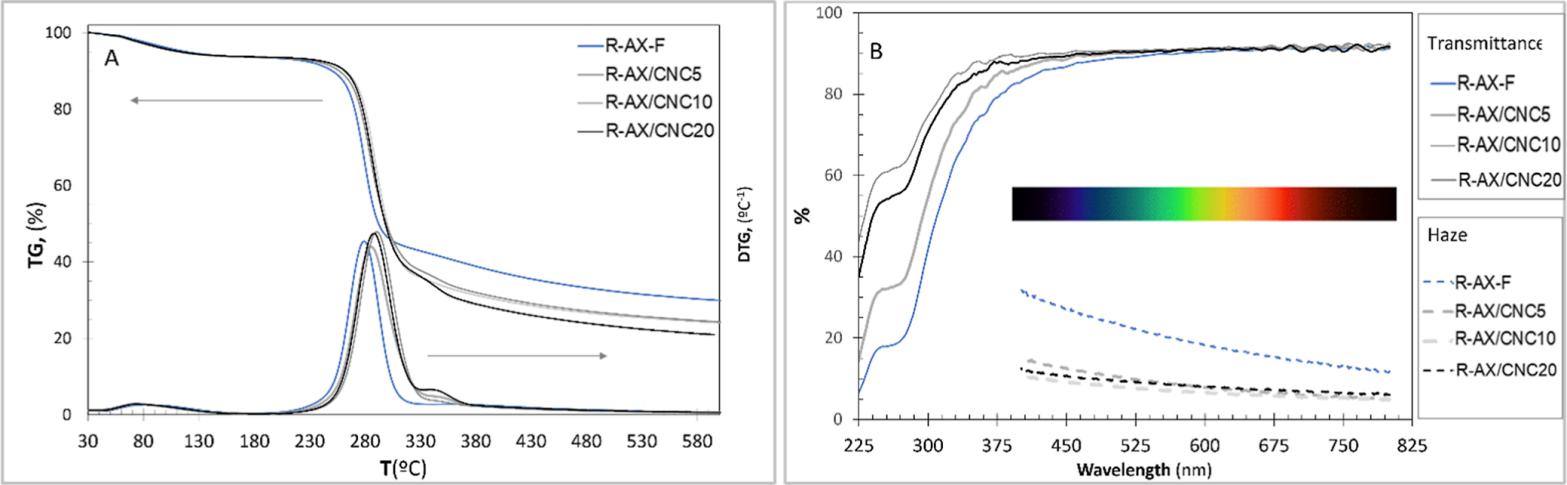
Effect of RB-CNC content on the (A) thermogravimetric (TG) and derivative thermogravimetric (DTG) curves and (B) UV/vis spectra of rye AX-based films.

Figure 4B presents the UV–vis spectra of the films, enabling a quantitative evaluation of their UV-shielding performance and visible light transparency by assessing transmittance in the UV (220–400 nm) and visible (400–800 nm) ranges. The R-AX-F films exhibited moderate UV transmittance and high transparency in the visible region, which is an important attribute for food packaging, where visual inspection of the products is often desirable, and particularly important for edible fruit coatings, where enhanced transparency and reduced haze may also improve the fruit’s aesthetic appeal.^84^ UV-blocking efficiencies for R-AX-F reached up to 90% at 225 nm in the UV-C region, 80% at 280 nm in the UV-B region, and 50% at 350 nm in the UV-A region. This moderate UV-shielding performance may result from residual phenolic components and/or conjugated structures in AX formulations, which can absorb UV radiation.^85^ A progressive increase in transmittance was observed with increasing wavelength values until reaching 93% at 600 nm, which remained stable up to 800 nm. As RB-CNC content increased, the transmittance values increased, and therefore the wavelength at which the plateau was reached shifted to lower wavelengths. As a result, the R-AX/CNC10 and R-AX/CNC20 films maintained almost constant transmittance values of 93% across the visible spectrum, indicating high transparency across all color wavelengths.^85^ Therefore, the increasing addition of RB-CNC to AX-based composite formulations enhanced film transparency, suggesting a dense packing of macromolecular chains without cavities and a homogeneous dispersion of RB-CNC, minimizing undesired light scattering caused by nanoparticle aggregations.^86^

To further investigate the optical properties of the designed films, their transmission haze across different wavelengths was measured, and the results are displayed in Figure 4B. The R-AX/CNC10 and R-AX/CNC20 films displayed the greatest transparency, corresponding to the lowest haze values. The incorporation of 5% RB-CNC into the composite formulations resulted in an approximate 20% reduction in haze at 400 nm compared to neat R-AX-F. However, as the RB-CNC continued to increase, the reduction in haze became less significant. On the other hand, haze values decreased for each film as wavelengths increased; however, this trend was less pronounced in films with higher RB-CNC content. R-AX-F showed a 20% reduction in haze from 400 to 800 nm, whereas RAX/CNC20 films displayed only a 10% decrease over the same wavelength range, reaching a final haze value of 5%. All composite films not only exhibited higher transparency than neat R-AX-F, but also maintained exceptional visual clarity, with haze levels consistently remaining below 15% across the visible spectrum, rendering them highly suitable for transparent food packaging applications.^85^

## 4 Conclusions

This work introduces a novel biorefinery strategy for the valorization of an underutilized cereal bran, enabling the production of cellulose nanocrystals (RB-CNC) and their incorporation into all-rye-based arabinoxylan nanocomposite films. The RB-CNC isolation process was thoroughly monitored through an integrated comprehensive physicochemical analytical procedure of each biomass fraction obtained after sequential alkaline, bleaching, and hydrolytic treatments. This analysis revealed the gradual elimination of noncellulosic components and the transformation of cellulose from macro- to nanoscale structures. The resulting RB-CNC displayed very high purity (>90% cellulose), strong surface charge (−35.8 mV), an exceptional aspect ratio (∼61), and exceptional thermal stability (230 °C), making them highly suitable as biobased reinforcing agents.

Commercial rye arabinoxylan (R-AX) was selected as the polymeric matrix for the development of composite films containing 5%, 10%, and 20% RB-CNC. The neat R-AX films already exhibited competitive thermomechanical properties, optical transparency, and water vapor barrier performance to be used for transparent food packaging. The incorporation of RB-CNC further enhanced the mechanical strength, flexibility, thermal resistance, and optical transparency, while preserving the water vapor permeability characteristics of the neat R-AX-F. These improvements were attributed to synergistic interfacial interactions between the hydroxyl-rich surfaces of both RB-CNC and R-AX, which facilitated efficient load transfer and network cohesion within the film structure. Specifically, composite films with 10% RB-CNC achieved mechanical and thermal performance superior to that of commercial cellophane, while showing high optical transmittance (>92%) and low haze (<10%) across the visible spectrum, and WVP values up to four times lower than those of cellophane.

Overall, this study highlights rye bran as a value-added feedstock for CNC production within an integrated fractionation biorefinery framework and demonstrates the technical feasibility of using these CNC as reinforcing agents in the design of fully rye-based nanocomposite films for food packaging applications. These findings contribute to circular economy efforts by upgrading cereal processing side-streams into high-performance packaging materials and promoting more sustainable agro-industrial value chains. Nonetheless, the environmental impact of sulfuric acid hydrolysis must be carefully managed through acid recovery and effluent treatment, and future studies should include life cycle assessment (LCA) to fully evaluate the environmental trade-offs associated with CNC-based systems.
